# Women's Greater Ability to Perceive Happy Facial Emotion Automatically: Gender Differences in Affective Priming

**DOI:** 10.1371/journal.pone.0041745

**Published:** 2012-07-23

**Authors:** Uta-Susan Donges, Anette Kersting, Thomas Suslow

**Affiliations:** 1 Department of Psychosomatic Medicine, University of Leipzig, Leipzig, Germany; 2 Department of Psychiatry, University of Münster, Münster, Germany; Ecole Normale Supérieure, France

## Abstract

There is evidence that women are better in recognizing their own and others' emotions. The female advantage in emotion recognition becomes even more apparent under conditions of rapid stimulus presentation. Affective priming paradigms have been developed to examine empirically whether facial emotion stimuli presented outside of conscious awareness color our impressions. It was observed that masked emotional facial expression has an affect congruent influence on subsequent judgments of neutral stimuli. The aim of the present study was to examine the effect of gender on affective priming based on negative and positive facial expression. In our priming experiment sad, happy, neutral, or no facial expression was briefly presented (for 33 ms) and masked by neutral faces which had to be evaluated. 81 young healthy volunteers (53 women) participated in the study. Subjects had no subjective awareness of emotional primes. Women did not differ from men with regard to age, education, intelligence, trait anxiety, or depressivity. In the whole sample, happy but not sad facial expression elicited valence congruent affective priming. Between-group analyses revealed that women manifested greater affective priming due to happy faces than men. Women seem to have a greater ability to perceive and respond to positive facial emotion at an automatic processing level compared to men. High perceptual sensitivity to minimal social-affective signals may contribute to women's advantage in understanding other persons' emotional states.

## Introduction

According to commonly held beliefs, women are more emotional, experiencing and expressing emotions in general more intensely than do men. They are also thought to have superiority in emotional competence such as understanding others' emotions [1], [2]. There is evidence from research using self-report and performance based measures that women have a better ability to identify and name their feelings than men even when controlling the effects of verbal intelligence, education, and socioeconomic status [3], [4].

An important aspect of emotional competence is the ability to recognize emotions from facial expressions. Facial expression of emotion is one of the most important types of signals encountered in interpersonal relations [5], [6]. Results from meta analyses show that there is a female advantage in recognizing facially expressed emotions even though the mean effect size seems to be rather small [7], [8]. Interestingly, gender differences become much more apparent when facial stimuli are presented at the edge of consciousness [9], [10]. Thus, women appear to recognize facial emotions better than men in particular under conditions of minimal stimulus information, i.e. when facial expression is shown for less than a second.

Facial electromyographic studies have revealed that when people are exposed to emotional facial expressions, they spontaneously react with distinct reactions in emotion-relevant facial muscles [11]. These reactions reflect a tendency to mimic the facial stimuli and occur even when facial expression is not consciously perceived [12]. It has been observed that women manifest more mimicking of facial emotional expression than men [13]. Moreover, women appear to display more emotional contagion to both positive and negative expressions than men in a dyadic interaction setting concerning subjective experience and facial response [14].

Zajonc [15] posited the existence of a fast-acting affective information-processing system to account for the fact (among others) that people often know their preference among several items before they can explain the reasons for that preference. Subsequently, experimental priming paradigms have been applied to examine empirically whether emotion-laden stimuli presented outside of conscious awareness color our impressions and judgments.

Murphy and Zajonc [16] exposed participants to subliminal facial expressions of emotions that varied in valence (angry, happy, and neutral) and were masked by ideographs. It was observed that valence of expression influenced judgments of subsequent ideographs. In the happy prime condition ideographs were evaluated more positively than those shown in the neutral prime condition, whereas in the angry prime condition ideographs were evaluated more negatively than those presented in the neutral control condition (for analogous findings, see Niedenthal [17]). This phenomenon of affect congruent influence of emotional facial expression on subsequent judgments is called *affective priming effect*. Subliminal affective priming is even found when participants are invited to attribute their emotional reaction to an alternative source, such as background music [18]. Furthermore, unconscious processing of facial emotions can influence consumption behavior [19] and can have rather long-lasting effects on memory [20].

Using facial electromyography during affective priming, Rotteveel et al. [21] found more frowning to ideographs preceded by subliminally presented angry than happy faces. It can be argued that facial mimicry occurs during subliminal processing of facial expression causing a matching emotional state in the observer which influences the evaluative response to the neutral mask stimulus.

The aim of the present study was to examine the extent of affective priming as a function of gender. To our knowledge, there exist no studies investigating the effect of gender on the automatic perception of facial expression using affective priming. In our priming experiment sad, happy, neutral, or no facial expression was briefly presented and masked by neutral faces. Subjects had to evaluate the neutral mask face. Since it has been shown that women are more responsive than men to facial emotions (especially when stimuli are perceived at the edge of consciousness) it was hypothesized that women would manifest stronger positive and negative affective priming effects compared with men.

## Methods

### Participants

Eighty-one young healthy volunteers (53 females) whose first language was German participated in this study which was conducted at the Department of Psychiatry of the University of Münster (see Table 1 for sociodemographic information). With regard to age and education, women did not differ from men (p>.05). All subjects were free of psychotropic medication and had normal or corrected-to-normal vision. They were screened to exclude any previous or current psychiatric, neurological, or medical diseases. Intelligence of study participants was assessed by the Multiple choice vocabulary test (MWT-B [22]). To measure participants' depressivity and trait anxiety the Beck-Depression Inventory (BDI [23]) and the State-Trait-Anxiety Inventory (STAI [24]) were administered (see Table 1 for details). No participant had a BDI score indicative of moderate or severe depression (>10). Women did not differ from men in depressivity, trait anxiety, or intelligence (p>.05). Subjects received a compensation of 10 EUR after their participation in the experiment.

**Table 1:** Sociodemographic characteristics, intelligence, and affectivity of study participants as a function of gender.

	Women (n = 53)		Men (n = 28)	
	Mean	SD	Mean	SD
Age (years)	24.4	5.8	26.1	8.2
Education (years)	12.6	0.9	12.5	1.3
Intelligence (IQ MWT-B)	117.1	12.4	115.7	14.8
STAI-Trait	33.3	6.9	34.6	9.2
BDI	3.0	3.7	2.5	2.8

MWT-B: Multiple choice vocabulary test; STAI-Trait: State-Trait Anxiety Inventory, trait version; BDI: Beck Depression Inventory.

### Ethics statement

Our experiment was carried out according to the Declaration of Helsinki. The study was approved by the ethics committee at the University of Münster. Written informed consent was obtained from all subjects.

### Stimulus material and procedure

Facial stimuli in the affective priming experiment consisted of grey-scale normalized sad, happy, and neutral expressions taken from the Facial Emotion Discrimination Test [25]. Emotional and neutral faces of 10 individuals (50% female and 50% male, respectively) were used as primes. Vertically mirrored neutral faces of the same individuals were used as masking stimuli. 40 trials were shown: 10 with sad, 10 with happy and 10 with neutral prime faces; in 10 trials no prime faces were presented. In each trial containing two faces facial expressions of the same individual were displayed. Faces were shown in a fixed random sequence with the restriction of no more than one repetition of a prime condition on consecutive trials. Each trial had a duration of 10 seconds: a prime face was shown for 33 ms preceded by a fixation cross for 1000 ms and followed by a neutral face for 967 ms. It followed a blank screen for 8 seconds. In this time period subjects had to evaluate the briefly shown neutral (mask) face as expressing rather negative or rather positive feelings by pressing one of six buttons (−2,5, −1.5, −0.5, +0.5, +1.5, +2.5) on a keyboard. Participants were told that the faces had slight differences concerning their affective expressions. The affective priming experiment had an overall duration of 6 minutes and 40 seconds.

Three affective priming scores were computed by subtracting mean evaluative ratings for neutral mask faces primed by no faces from mean evaluative ratings for neutral mask faces primed by happy (sad or neutral) faces (mean evaluative ratings for each study group are presented in Table 2). A negative priming score for sad faces indicates that subjects rated the neutral masks more negatively if they were primed by sad faces, compared with neutral masks primed by no faces. A positive priming score for happy faces indicates that subjects evaluated neutral masks more positively if they were primed by happy faces, compared with neutral masks primed by no faces (see Table 3 for priming scores).

**Table 2:** Evaluative responses to neutral mask faces as a function of prime and gender.

	Women (n = 53)		Men (n = 28)	
	Mean	SD	Mean	SD
Sad prime condition	−0.144	0.255	−0.045	0.186
Happy prime condition	0.015	0.250	−0.070	0.168
Neutral prime condition	−0.103	0.254	−0.069	0.178
No prime condition	−0.093	0.270	−0.061	0.181

**Table 3:** Affective priming scores based on sad, happy, and neutral primes as a function of gender (baseline condition: no prime).

	Women (n = 53)		Men (n = 28)	
	Mean	SD	Mean	SD
Sad prime	−0.051	0.255	0.016	0.213
Happy prime	0.108	0.244	−0.010	0.127
Neutral prime	−0.010	0.148	−0.008	0.192

### Awareness check

To assess the success of masking emotional facial expression subjects were thoroughly questioned immediately after the priming experiment as to whether they had noticed anything out of the ordinary and whether they had seen anything just before the neutral faces. None of the participants reported to have noticed emotion faces or emotional expression before the neutral faces. The subjective threshold is the oldest criterion for demonstrating perception without awareness and is attractive because it directly assesses the conscious experience of subjects [26].

## Results

There was a general tendency to evaluate neutral mask faces on average as rather negative. In the whole sample, the mean priming score based on sad faces was −0.028 (SD  = 0.242), mean priming score based on happy faces was 0.067 (SD  = 0.218), and mean priming score based on neutral faces was −0.009 (SD  = 0.164). According to paired two-sample *t*-tests the priming score based on happy faces differed significantly from the priming scores based on neutral and sad faces (*t*(80)  = 3.00, *p*<.005; *t*(80)  = 3.03, *p*<.005). The priming scores based on sad and neutral faces did not differ from each other (*t*(80)  = −0.72, *p* = .47). Thus, in the whole sample there was evidence for a valence-congruent judgmental shift due to masked happy faces.

Correlation analyses for the total sample showed that affective priming scores were not related to depressivity (BDI), trait anxiety (STAI), age, or education. There was only a significant correlation between affective priming due to sad facial expression and intelligence (*r* = −.26, *p*<.05).

The affective priming data were further analyzed in a repeated measures analysis of variance (ANOVA), with one between-subjects factor (gender: female vs. male) and one within-subjects factor (prime face: happy, sad, and neutral). The effect of prime face reached marginal significance (*F*(2, 78)  = 2.83, *p* = .06). The effect of gender was not significant (*F*(1, 79)  = 0.72, *p* = .40). The interaction between prime face and gender was significant (*F*(2, 78)  = 5.15, *p*<.01). The results from unpaired two sample *t*-tests showed that women did not differ from men in affective priming due to sad or neutral facial expression (*t*(79)  = −0.80, *p* = .43; *t*(79)  = −0.09, *p* = .93). However, there was a significant between-group difference for affective priming based on happy faces (*t*(79)  = 3.47, *p*≤.005). Thus, women exhibited greater affective priming due to happy faces than men.

Paired two-sample *t*-tests calculated in the male sample show that the priming score based on happy faces did not differ from the priming scores based on neutral or sad faces (*t*(27)  = −0.44, *p* = .66; *t*(27)  = −0.71, *p* = .48). The priming scores based on sad and neutral faces did also not differ from each other (*t*(27)  = 0.21, *p* = .84). Thus, in the male sample there was no evidence for valence-congruent evaluative shifts due to masked happy or sad faces. For women, the priming score based on happy faces was significantly different from those based on neutral or sad faces (*t*(52)  = 3.81, *p*<.001; *t*(52)  = 4.01, *p*<.001). The priming scores based on sad and neutral faces did not differ from each other (*t*(52)  = −0.99, *p* = .33).

Response latencies in the affective priming experiment were analyzed as a function of study groups and prime conditions (see Table 4 for response latencies). A repeated measures analysis of variance (ANOVA), with one between-subjects factor (gender: female vs. male) and one within-subjects factor (prime condition: happy, sad, neutral, no-face) yielded no significant effects of gender (*F*(1, 79)  = 0.41, *p* = .52), prime condition (*F*(3, 77)  = 1.25, *p* = .30) or interaction of gender and prime condition (*F*(3, 77)  = 1.32, *p* = .27). Thus, response time did not vary as a function of prime condition and gender.

**Table 4:** Response latencies (in ms) to neutral mask faces as a function of prime and gender.

	Women (n = 53)		Men (n = 28)	
	Mean	SD	Mean	SD
Sad prime condition	1.405	302	1.420	340
Happy prime condition	1.357	292	1.414	332
Neutral prime condition	1.361	297	1.432	345
No prime condition	1.386	296	1.424	332

## Discussion

In the present investigation affective priming based on facial expressions of emotions was examined for the first time as a function of gender. The affect congruent influence of briefly presented emotional facial expression on subsequent judgments has been shown repeatedly in previous research [16]–[18], [27]. In our priming experiment we investigated the evaluative shifts elicited by negative (sad) and positive (happy) facial expression shown below the threshold of subjective conscious perception. According to an interview conducted immediately after the priming experiment, study participants had no subjective awareness of emotional primes. Confirming our hypotheses at least in part, the present results suggest that there exist gender differences in affective priming due to happy facial expression. Women showed a significantly stronger positive evaluative shift in the happy prime condition than men. It appears that women have a greater ability to perceive and respond to positive facial emotions at an automatic processing level. Interestingly, the phenomenon of affect congruent influence of happy facial expression on subsequent judgments was observed only for women. In the male group, no evidence for affective priming due to emotional primes was observed. Thus, the significant affective priming effect for happy faces found in the whole sample was the result of women's prime-valence congruent evaluative shifts. In view of our findings, it appears advisable to control gender effects in future studies on affective priming.

According to our results there were no gender differences in affective priming elicited by sad facial expression but there was also no affective priming effect for sad faces in the whole sample. Future research has to clarify whether sad compared to threat-related (i.e. angry or fearful) facial expression produces weaker valence-congruent judgmental shifts. Previous studies on affective priming have preferentially used angry (and happy) faces as prime stimuli [16], [21]. It has been shown that facial expression of sadness is perceived as less arousing compared to facial expressions of anger or happiness [28]. Thus, it remains to be examined in future studies whether high-arousal facial emotions (e.g., angry faces) lead to stronger affective priming effects compared to low-arousal emotional expressions such as sad faces.

The present results are consistent with the observation of a female advantage in recognizing facially expressed emotions especially under rapid presentation conditions [9], [10]. Our findings indicate that women appear to recognize positive valence of facial emotion even when it is processed outside of conscious awareness. According to Rotteveel et al. [21] subtle mimicking responses occur during affective priming. It is known that tendencies to imitate facial expression takes place even when it is not consciously perceived [12]. Women have been found to manifest more mimicking of facial emotional expression [13] and to display more emotional contagion during dyadic interaction than men [14]. It appears plausible that women's superiority in recognizing and responding automatically to facial emotion could be related to an enhanced mimicking response. Affective simulation involving also facial mimicking seems to provide a direct understanding of the emotions felt by others [29]. Preventing facial mimicry, which is presumably subserved by the mirror neuron system, can impair the identification of facial emotions [30]. Recent fMRI data [31] suggest that females recruit areas containing mirror neurons to a higher degree than males in face-to-face interactions. Thus, stronger responses of an affective mirror neuron system may underlie enhanced affective priming and facilitated emotional contagion in females.

Since only a subjective awareness check was conducted in our study no strong conclusions can be drawn about unawareness of emotion primes. We do not claim to have assessed subliminal perception of facial emotions in our experiment. The administration of an objective awareness task would have allowed more definite conclusions about participants' awareness of prime stimuli. However, insofar as the duration of prime presentation was very short (i.e., the stimulus onset asynchrony was 33 ms) our affective priming experiment should have measured fast evolving automatic responses to facial emotions in our female sample that probably do not require effort or intention. According to recent models of information processing automaticity is not a unitary construct [32]. It can be diagnosed by considering the presence of different features such as fast, unintentional, uncontrollable, unconscious, and efficient. Not all of these features or conditions have to be fulfilled to assume automaticity [33], [34]. In our experiment, women's processing of masked happy facial expression should have been automatic in the sense of fast, unintentional, and efficient. Subjects had no subjective awareness of emotional primes so that it appears very unlikely that they had conscious intent to process the happy (or sad) faces. The processing of the happy faces should have occurred under conditions of inattention or distraction. Moreover, women's processing of happy faces should have been efficient in our experiment. A process is defined as efficient when it needs little (or no) processing resources [35]. In our female sample, evaluation latencies in the happy prime condition were lower than those in all other prime conditions. Thus, the positive evaluative shifts elicited by the processing of masked happy facial expression seem not to be associated with reaction time costs.

Recently, Gohier et al. [35] reported evidence for a greater sensitivity to negative emotional stimuli in healthy women compared to healthy men in a cross-modal affective priming experiment in which primes were clearly visible (i.e. the stimulus-onset asynchrony between prime and target was 400 ms). These findings appear to be at odds with our own results indicating stronger affective priming in women compared to men for positive (happy) but not negative (sad) faces. There are several differences in stimulus material, presentation duration, and dependent variables between the studies that might explain the discrepant findings. By administering clearly visible emotional faces, sounds, and words as primes Gohier et al. [35] should have measured primarily controlled or explicit processing, whereas in our study automatic processes were assessed. Moreover, Gohier et al. examined priming defined as reaction time facilitation, while we investigated priming effects in the sense of evaluative shifts. Finally, Gohier et al. used angry and fearful facial expressions as negative stimuli, whereas in our study sad faces were presented. Gohier et al. 's results suggest a greater sensitivity to negative, threat-related stimuli in women compared to men at a controlled processing level. Thus, when stimuli are clearly visible women appear to allocate more attention to threat-relevant stimuli than men. According to our results, women seem not more sensitive to facial expressions of sadness than men at an automatic processing level which does not contradict Gohier et al. 's findings. Sadness signals helplessness or unavailability of the expresser [36]. Sadness as an appeal for caretaking is in general evaluated as less threatening than anger which implies a critique and signals the necessity to handle conflict [37]. Future investigation is needed to clarify whether women show stronger evaluative shifts due to masked angry or fearful faces than men.
